# Genome Mining of *Cronobacter sakazakii* in Bangladesh Reveals the Occurrence of High-Risk ST83 and Rare ST789 Lineages

**DOI:** 10.3390/pathogens14121220

**Published:** 2025-11-30

**Authors:** Sutapa Bhowmik, Supantha Rivu, Md. Latiful Bari, Sangita Ahmed

**Affiliations:** 1 Department of Microbiology, Noakhali Science and Technology University, Noakhali 3814, Bangladesh; 2 Department of Microbiology, University of Dhaka, Dhaka 1000, Bangladesh; supantharivu008@gmail.com; 3 Department of Microbiology, Notre Dame University Bangladesh, 2/A, Arambagh, Motijheel, Dhaka 1000, Bangladesh; 4 Food, Nutrition and Agriculture Research Division, Centre for Advanced Research in Sciences, University of Dhaka, Dhaka 1000, Bangladesh

**Keywords:** *Cronobacter sakazakii*, powdered infant formula, mobile genetic elements, virulence, antibiotic resistance genes, CRISPR-cas

## Abstract

*Cronobacter sakazakii* is a foodborne pathogen of major concern due to its link with severe neonatal infections through powdered infant formula (PIF). However, its genomic epidemiology in Bangladesh remains uncharacterized. We report the first whole-genome analysis of three isolates from PIF. Two isolates (S41_PIFM and S44_RUTF) belonged to ST83, a lineage repeatedly associated with neonatal meningitis, septicemia, and persistence in PIF production environments, while the third (S43_TF) represented ST789, a recently described and rare lineage of unknown pathogenic potential. Pan-genome and comparative analyses identified 39 virulence determinants, 19 antimicrobial-resistance genes, and diverse mobile genetic elements. ST83 isolates harbored plasmid replicons IncFII(pCTU2) and pESA2, while the ST789 isolate carried insertion sequence ISKpn34, indicating horizontal gene transfer potential. All strains encoded I-E CRISPR-Cas systems. The detection of globally recognized high-risk ST83 clones alongside the novel ST789 lineage highlights emerging public health risks. This study provides the first genomic insights into *C. sakazakii* in Bangladesh and underscores the urgent need for genomic surveillance and strengthened food safety monitoring to protect infant health in low- and middle-income countries.

## 1. Introduction

The genus *Cronobacter* is a group of opportunistic Gram-negative pathogens belonging to the *Enterobacteriaceae* family that consists of seven species: *C. sakazakii*, *C. malonaticus*, *C. turicensis*, *C. universalis*, *C. condiment*, *C. dublinensis*, and *C. muytjensii* [1,2]. Among them, *C. sakazakii* (previously known as *Enterobacter sakazakii*) and *C. malonaticus* cases are associated with human infections in all age groups, with some rare instances of *C. turicensis* and *C. universalis* [3,4]. *Cronobacter*-mediated infections are more frequent in the younger (<14 years) and older populations (>65 years) than the adults (15–65 years), with the highest incidence rate in infants [5]. The mortality rate of this pathogen ranges from 15 to 80%, and the clinical profile is primarily meningitis, septicemia, or necrotizing enterocolitis [6,7].

Several virulence factors have been linked to this pathogen’s ability to cause infection. These virulence factors are linked to motility, cell adhesion and invasion, survival in macrophages, sialic acid utilization, capsule formation, and endotoxin production. The primary virulence genes associated with these features include *ompA*, *cpa*, *fliC*, *hly*, *sip*, *aut*, *plas*, and *inv* [8]. Moreover, persistence of *C. sakazakii* across various physicochemical parameters, ability to form biofilms, and resistance to antibiotics all add to its pathogenicity potential.

Although *C. sakazakii* has been isolated from various food products such as mixed salad vegetables, meat, milk, and cheese, contaminated powdered infant formula (PIF) stands out as a major source of infection in infants [9,10]. The contamination of the PIF could be either intrinsic or extrinsic contamination of utensils and processing equipment [11]. In addition, *Cronobacter* spp. have been found up to 24 months after the PIF was packaged, implying that they survive in this food product and pose a risk to infant health [12]. However, the incidence of *C. sakazakii* in PIF varies significantly by region, with North America reporting the highest levels (38.77%), followed by South America (18.12%), Africa (13.00%), Asia (7.59%), and Europe (5.45%) [13]. Furthermore, whole genome sequencing (WGS)-based characterization of *C. sakazakii* from multiple sources revealed a diverse genetic profile. The most frequently identified *C. sakazakii* in PIF sold in various nations’ PIF manufacturing facilities include ST4, ST1, and, to a lesser extent, ST83. These sequence types are also common in invasive clinical conditions, including septicemia and meningitis. As a result, an in-depth WGS study of *C. sakazakii* can aid in determining its virulence potential, pathogenicity, and potential treatment approaches.

In Bangladesh, one *Cronobacter* sp. was first identified in 36 PIF samples, and later six *C. sakazakii* isolates were found, three from powdered milk samples, one from Horlicks, biscuits, and spices samples [14,15]. These studies identified antimicrobial resistance, biofilm formation, stress tolerance, and virulence genes (*ompA* and *zpx*) in *C. sakazakii* isolates. However, no genome characterization data for any *C. sakazakii* isolate from Bangladesh are available, which could shed light on the pathogen’s circulating genotypes, transmission, or epidemiological relationship in the country. Therefore, this study performs a comprehensive analysis of the whole genome sequences of three *C. sakazakii* isolates obtained from PIF in Bangladesh, with a particular emphasis on pathogenicity, persistence, antibiotic resistance, phages, and mobile genetic elements. Furthermore, all research isolates were compared with worldwide isolates to identify potential epidemiological linkages.

## 2. Materials and Methods

### 2.1. Isolation and Identification of the C. sakazakii Isolates

A total of 65 samples were collected, including powdered infant formula milk (61), ready-to-use therapeutic food (2), and therapeutic food (2). For the isolation of *C. sakazakii*, 25 g of each sample was enriched in sterile 225 mL buffered peptone water (BPW). Following overnight incubation at 37 °C, 10 mL of the enriched sample was inoculated into 90 mL of Enterobacteria Enrichment (EE) broth and was incubated at 37 °C for 24 h. A loopful of the culture from EE broth was inoculated on *Enterobacter sakazakii* agar (ESA), followed by incubation at 37 °C for 48 h. The *C. sakazakii* isolates were presumptively identified based on their morphology on ESA. Presumptive isolates were identified as *Cronobacter* spp. by detection of the genus-specific *gluA* gene (EsAgf 5′-TGA AAG CAA TCG ACA AGA AG-3′ and EsAgr 5′-ACT CAT TAC CCC TCC TGA TG-3′), and amplification of the *Cronobacter*-specific 16S rRNA gene (Esak2 5′-CCC GCA TCT CTG CAG GAT TCT C-3′ and Esak3 5′-CTA ATA CCG CAT AAC GTC TAC G-3′) [16,17].

### 2.2. DNA Extraction and Whole Genome Sequencing

Using the Qiagen DNeasy Blood and Tissue Kit (250) (Qiagen, #69504, Hilden, Germany), genomic DNA of each isolate was extracted from Luria broth (Oxoid, Basingstoke, Hampshire, UK). After checking the quality of the extracted DNA using Nanodrop (Thermo Fisher Scientific, ND-1000, Waltham, MA, USA) and Qubit (Thermo Fisher Scientific, Q33238, Waltham, MA, USA), DNA samples were sent to the International Center for Diarrheal Disease Research, Dhaka, Bangladesh (ICDDR, B) for whole genome sequencing utilizing the Illumina NextSeq 2000 platform (Illumina, San Diego, CA, USA). For library preparation, the Illumina DNA Prep Reagent Kit (20060059) (Illumina, San Diego, CA, USA) was used alongside an automated liquid handler (epMotion 5075). Post-pooling, the library concentration was quantified using a Qubit 4.0 fluorometer, ensuring it was adequate for sequencing. The sequencing process involved the use of fluorescently labeled nucleotides to decode the DNA sequence from the prepared libraries, utilizing paired-end 2 × 150 bp reads to capture detailed genetic data.

### 2.3. Genome Sequence Assembly, Identification, and Annotation

For each isolate, two fastq files were uploaded to the Galaxy server, and the FastQC software (version 0.74) was used to check their quality. Followed by adapter trimming by Trim Galore v0.6.10, the fastq files were assembled into a single fasta file using SPAdes (version 4.2.0) [18]. The assembled genomes’ quality was determined by QUAST (version 5.3.0) [19]. Contigs ≥ 500 bp were selected for further analysis. Identification of the isolates was performed by utilizing public databases for molecular typing and microbial genome diversity (PubMLST) [20]. The sequence type of the isolates was determined using MLST 2.0.9 on the Center for Genomic Epidemiology [21]. The *gnd* and *galF* loci gene clusters, specific for the O-serotype region, were identified from the BLAST gDNA sequences by the BIGSdb pipeline tools in the PubMLST typing database (pubmlst.org/organisms/cronobacter-spp (15 August 2025)) [22,23]. Rapid Annotation using Subsystem Technology (RAST) web server and Prokaryotic genome annotation (Prokka) (Galaxy version 1.14.6) were utilized for genome annotation [24,25].

### 2.4. Phylogenetic and Pan-Genome Analysis

To compare the study isolates with different species of *Cronobacter* and a few species from other closely related genera, a phylogenetic tree was constructed in Type Strain Genome Server (TYGS) [26]. The tree was inferred with FastME 2.1.6.1 [27] from Genome BLAST Distance Phylogeny (GBDP) distances calculated from genome sequences. The branch lengths were scaled in terms of GBDP distance formula d5. The tree was rooted at the midpoint. Later, the BLAST Ring Image Generator (BRIG) tool v0.95 was used to compare the genomes. *C. sakazakii* NBRC 102416 (NCBI accession NZ_BAWU00000000) was used as a reference genome for this purpose [28].

For pan-genome analysis, all the complete genomes of *C. sakazakii* with proper assembly accession (*n* = 18) available in the Bacterial and Viral Bioinformatics Resource Center (BV-BRC) were downloaded (Supplementary Table S1). The pan-genome analysis was performed on the study sequences (*n* = 3) and the downloaded sequences with Integrated Prokaryotes Genome and pan-genome Analysis (IPGA) v1.09 [29]. Roary was chosen for genome clustering and pan-genome analysis processes [30].

### 2.5. Detection of Antimicrobial Resistance (AMR) and Virulence Genes, Mobile Genetic Elements, and Other Characterization

The Resistance Gene Identifier (RGI, v6.0.5) in Comprehensive Antibiotic Resistance Database (CARD, v4.0.1) was used to detect AMR genes, with the default “perfect” and “strict” settings [31]. PathogenFinder 1.1 was utilized to determine the pathogenic potential of the isolates towards humans [32]. ABRicate tool (version 1.0.1) was used to identify virulence genes against the virulence factor database (VFDB) [33]. For VFDB, the %coverage and %identity were set to 80 and 70, respectively. Enterobacteriales (database version 2023-01-18) on PlasmidFinder (software version 2.0.1, 2020-07-01) on the Center for Genomic Epidemiology was applied to detect plasmid replicons [34]. To find CRISPR arrays and their associated Cas proteins, CRISPRCasFinder v4.2.20 was utilized, while PHAge Search Tool with Enhanced Sequence Translation (PHASTEST) was used to identify prophage sequences in the genomes [35,36]. The prophage region was considered intact when the score was above 90, questionable if the score was between 70 and 90, and incomplete if the score was below 70. MobileElementFinder software (version 1.0.3) and ISfinder database (version 1.0.2) on the Center for Genomic Epidemiology platform were applied to determine the presence of mobile genetic elements (MGEs) [37]. In addition, antiSMASH 8.0 was applied to detect secondary metabolite biosynthesis gene clusters in our isolates [38]. Default parameters were used for all software unless otherwise specified.

## 3. Results

### 3.1. General Features of the Isolated C. sakazakii Strains

The draft assembly sizes of the three isolates varied from 4,366,115 to 4,444,405 bp, and the GC contents ranged from 56.84 to 56.98% (Table 1). While the L50 ranged from 5 to 23, the N50 value varied from 60,688 to 272,134 bp (Table 1).

### 3.2. Identification and Genome Annotation

**Figure 1:**
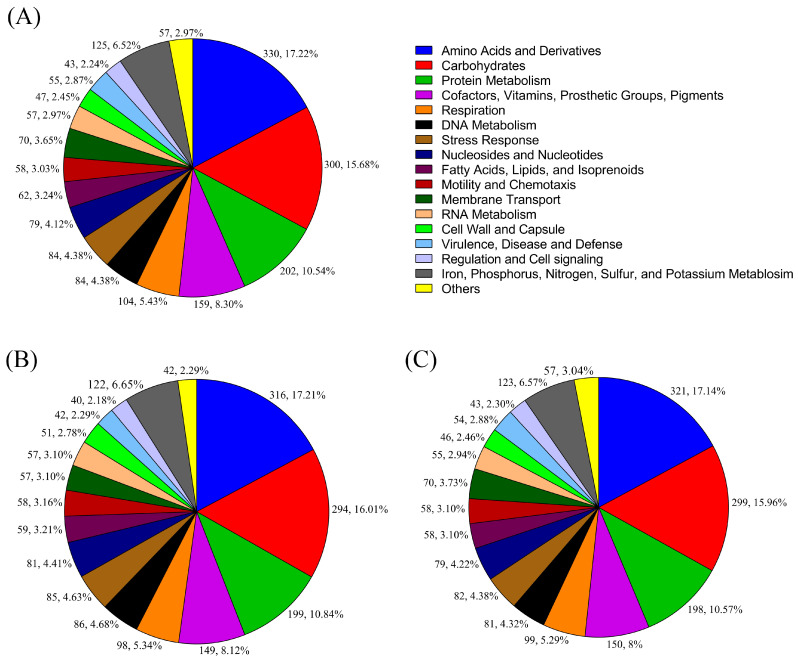
Distribution of the features in subsystems in the *C. sakazakii* genomes by RAST; (**A**) S41_PIFM, (**B**) S43_TF, and (**C**) S44_RUTF.

PubMLST confirmed all three isolates as *C. sakazakii* with 100% confidence. Annotation revealed the presence of almost similar numbers of protein-coding sequences (CDS) ranging from 4037 to 4076, 3–5 rRNA, and 1 tmRNA in the genomes (Table 2). Proteins with functional assignments also varied between 3596 and 3713. RAST showed that all the genomes had almost the same number of subsystems (Table 2). The highest number of features observed in the subsystems of every genome was related to amino acids and derivatives, followed by carbohydrates and protein metabolism. The features in the subsystems did not vary that much (Figure 1).

MLST identified one isolate, S43_TF, as ST789, and the other two, S41_PIFM and S44_RUTF, were designated as ST83. The complete MLST profiling is summarized in Table 3. Additionally, the S43_TF displayed a different clonal complex, CC13, while the other two belonged to CC83. The O-serotype analysis revealed that the ST83 *C. sakazakii* isolates (S41_PIFM and S44_RUTF) from ready-to-use therapeutic food and powdered infant formula milk, respectively, were representatives of O-antigen serotype (Csak_O3) gene clusters located between the *gnd* and *galF*, whose loci were *galF* 21 and *gnd* 65. The S43_TF isolate from therapeutic food represented an unknown serotype, whose loci were *galF* 235 and *gnd* 236.

### 3.3. Phylogenetic and Pan-Genome Analysis

**Figure 2:**
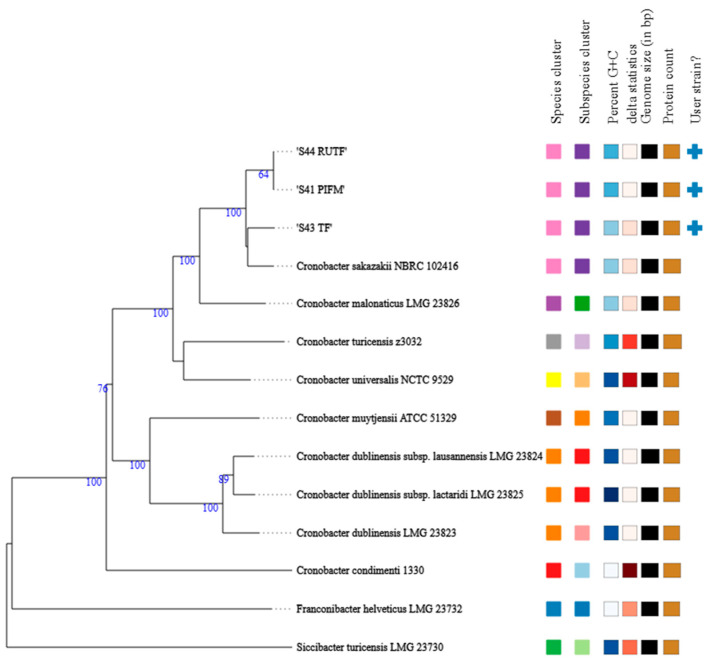
The phylogenetic tree shows the closest relation of the study isolates to *C. sakazakii*. The numbers above those branches are GBDP pseudo-bootstrap support values > 60% from 100 replications, with an average branch support of 85.3%.

**Figure 3:**
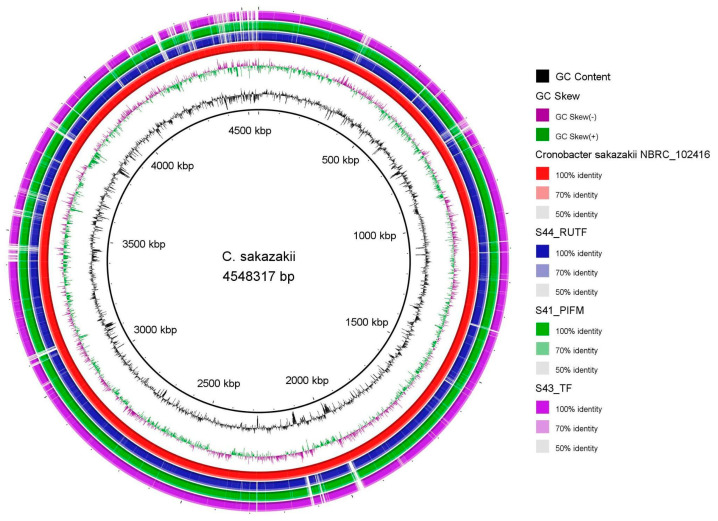
BRIG shows a comparison among the genomes. Here, *C. sakazakii* NBRC 102416 was used as a reference genome.

Based on TYGS analysis, all the study isolates were in the same group and had the closest relationship with *C. sakazakii* NBRC 102416, a type strain for *C. sakazakii* (Figure 2). A genome comparison among the three isolates, using BRIG and *C. sakazakii* NBRC 102416 as a reference, demonstrated some variations. While the isolates S41_PIFM and S44_RUTF were very similar, with only a few variations between them, S43_TF displayed different genomic features from the other two isolates (Figure 3).

**Figure 4:**
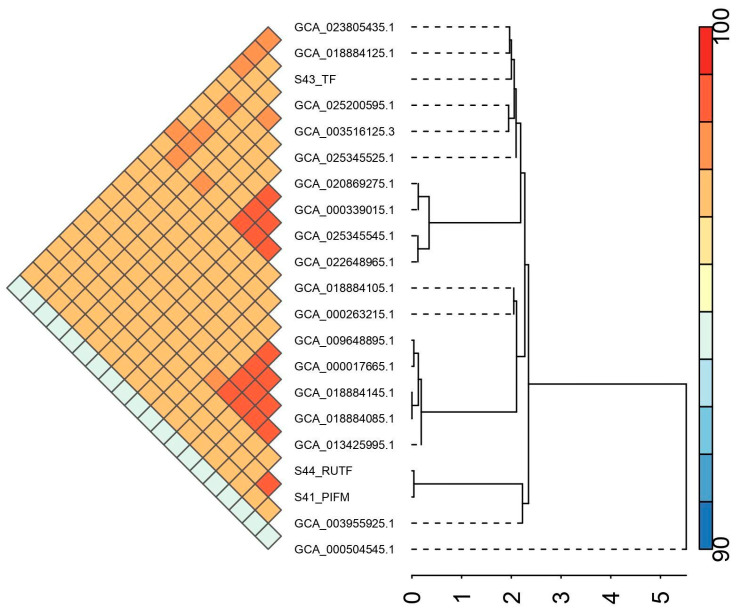
ANI-based relationship among the isolates in pan-genome analysis.

Average nucleotide identity (ANI) based analysis showed that the study isolates and downloaded complete genomes from BV-BRC (*n* = 18) were similar to each other with ANI values ranging from 94.48 to 99.96 (Supplementary Table S2). Phylogenetic analysis revealed that S44_RUTF and S41_PIFM were closest to *C. sakazakii* GZcsf_1 (GCA_003955925.1) obtained from brain abscess fluid in China, while S43_TF was closely related to *Cronobacter sakazakii* C105731 (GCA_018884125.1, alfalfa sprouts, Mexico) and *Cronobacter sakazakii* JXES_28 (GCA_023805435.1, infant food, China) (Figure 4).

**Figure 5:**
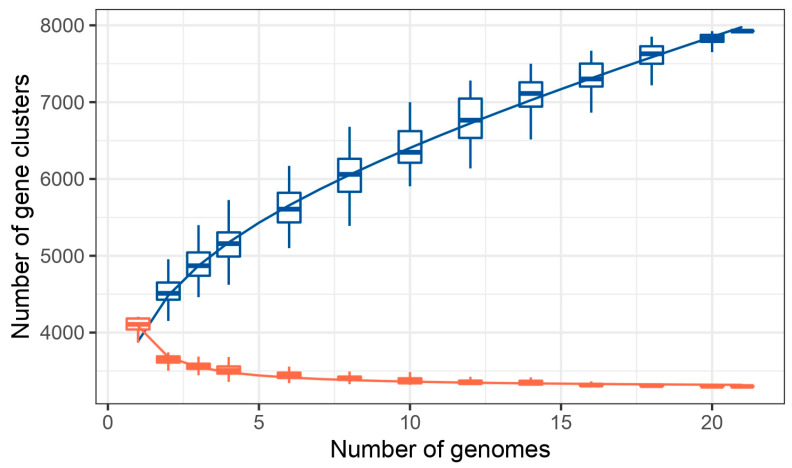
Distribution of core (orange) and accessory genes (blue) in pan-genome analysis.

Pan-genome analysis of the study isolates, along with *C. sakazakii* strains available in the NCBI database (*n* = 18), showed that these genomes share 3317 core genomes and a total of 7903 genes. Based on this analysis, 20, 84, and 9 unique genes have been identified in the isolates from the current study, S41_PIFM, S43_TF, and S44_RUTF, respectively (Table 4). Some of the identified unique genes play diverse roles in the survival and adaptability of the organism. Metabolism-related genes in S44_RUTF, *astE* (succinylglutamate desuccinylase), and *curA* (curcumin reductase) aid nitrogen metabolism and protect against oxidative stress, respectively. Another gene found in the same genome, *nasR*, helps adapt to low-oxygen conditions via nitrate regulation. There were four genes in S41_PIFM, *aplIM*, *xerC_4*, *hin*, and *jefA*, which have a role in the defense mechanism of the bacterium. *aplIM* (restriction-modification methylase), *xerC_4* (tyrosine recombinase), and *hin* (DNA-invertase) have a role in phage protection, chromosome segregation, and plasmid stability, and phase variation, respectively, which enhance immune evasion. In addition, the *jefA* gene plays a role as a drug efflux pump. Moreover, in S43_TF, two genes, *fdhF_5* and *pepP*, were found. *fdhF_5* aids anaerobic energy generation through formate oxidation, and *pepP* facilitates protein degradation for nutrient acquisition (Table 5). The core/pan rarefaction curve demonstrated that the number of pan-genome genes increased steadily with the addition of new strains, whereas the core genome exhibited the opposite trend (Figure 5). Since there was no obvious plateau in the core/pan-genome ratio, it indicates that the *C. sakazakii* pan-genome was in an open state.

### 3.4. Antimicrobial Resistance Genes

CARD detected a total of 19 distinct AMR genes in the three isolates, including *acrA*, *adeF*, *cRP*, *emrB*, *emrR*, *H-NS*, *kpnE*, *kpnF*, *msbA*, *qacG*, *qacJ*, *rsmA*, *marA*, *csa1*, *fosA8*, *vanG*, *eF-Tu*, *pbp3*, and *acrAB-tolC*. Except for *qacG*, *qacJ*, and *csa1*, all genes were shared by S41_PIFM, S44_RUTF (both ST83), and S43_TF (ST789). S43_TF lacked the previously mentioned genes. Among these, the *csa1*, and *fosA8* gene products inactivate antibiotics, while the *vanG*, *eF-Tu*, and *pbp3* genes are associated with alterations of the target molecule. *acrAB-tolC* can both alter the antibiotic target and perform antibiotic efflux. The rest of the detected genes encode proteins that are associated with efflux mechanisms to confer antibiotic resistance (Table 6). Among the genes, a few confer resistance against a single class of antibiotics. For instance, *emrB* and *emrR* (fluoroquinolones), *eF-Tu* (elfamycins), *pbp3* (beta-lactams), *msbA* (nitroimidazoles), *csa1* (cephalosporins), *fosA8* (phosphonic acids), *vanG* (glycopeptides). On the contrary, *acrA*, *adeF*, *cRP*, *H-NS*, *kpnE*, *kpnF*, *rsmA*, and *marA* function against diverse classes of antibiotics, including fluoroquinolones, cephalosporins, glycylcyclines, penicillin beta-lactams, tetracyclines, rifamycins, phenicols, marcolids, disinfecting agents, and antiseptics. In particular, the *qacG* and *qacJ* genes are specifically involved in resistance towards disinfecting agents and antiseptics (Table 6, Supplementary Table S3).

### 3.5. Virulence Factors

All three isolates were predicted as human pathogens with a probability from 0.799 to 0.802 by PathogenFinder (Supplementary Table S4).

**Figure 6:**
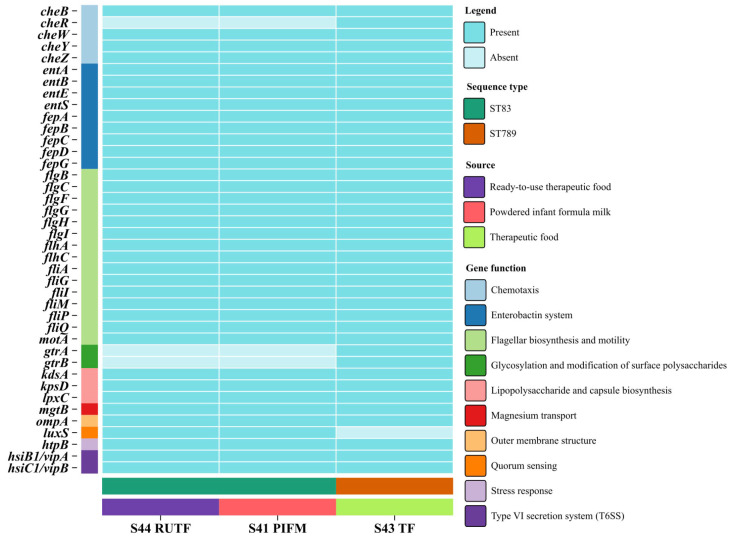
Overview of the virulence factors in the study genomes. Here, the color strip on the y-axis (left) represents the gene function, while on the x-axis, the upper strip represents sequence type and the lower strip represents the source of the isolate.

The three isolates S44_RUTF, S41_PIFM, and S43_TF exhibited a largely similar pattern of virulence genes, with some variations. Isolates S44_RUTF and S41_PIFM each carried 37 virulence genes, while S43_TF had 39 genes (Figure 6). The largest group of genes (15) was associated with flagellar biosynthesis and motility, including: *flgB*, *flgC*, *flgF*, *flgG*, *flgH*, *flgI*, *flhA*, *flhC*, *fliA*, *fliG*, *fliI*, *fliM*, *fliP*, *fliQ*, and *motA*. Among the others, nine genes were related to the enterobactin system (*entA*, *entB*, *entE*, *entS*, *fepA*, *fepB*, *fepC*, *fepD*, and *fepG),* three genes involved in lipopolysaccharide and capsule biosynthesis (*kdsA*, *kpsD*, and *lpxC*), two genes associated with the Type VI secretion system (*hsiB1/vipA*, and *hsiC1/vipB*), one gene for magnesium transport (*mgtB*), one stress response gene (*htpB*), and one gene encoding an outer membrane protein (*ompA*).

For several virulence genes, variation was observed among the three isolates. Isolate S43_TF carried five chemotaxis-related genes: *cheB*, *cheR*, *cheW*, *cheY*, and *cheZ*. All these genes, responsible for catalyzing the methylation of the cytosolic signaling domain of chemoreceptors, were present in the other two isolates (S44_RUTF and S41_PIFM), except for *cheR*. In addition to that, the *gtrA* and *gtrB* genes, involved in the glycosylation and modification of surface polysaccharides, were absent in these two genomes but present in S43_TF. In contrast, the *luxS* gene, associated with quorum sensing, was detected in S44_RUTF and S41_PIFM, while absent in isolate S43_TF.

Additionally, antiSMASH detected in all three isolates the presence of aerobactin, a siderophore that chelates iron, and aids in bacterial virulence, and carotenoid, which can protect bacteria from oxidative immune defense in hosts.

### 3.6. Prophages, Plasmids, and Other Mobile Genetic Elements

**Figure 7:**
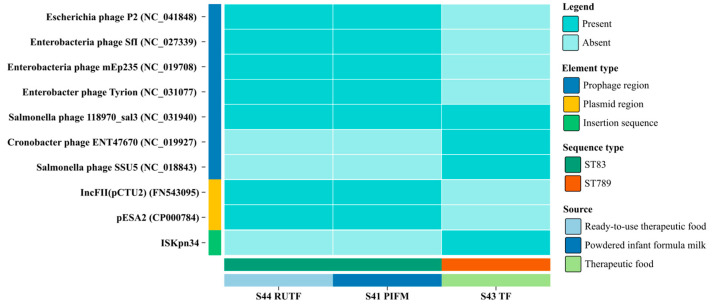
Overview of all the mobile genetic elements in the *C. sakazakii* isolates. Here, the color strip on the y-axis (left) represents the mobile genetic element type, while on the x-axis, the upper strip represents sequence type and the lower strip represents the source of the isolate.

Seven different types of prophages were identified in these three isolates. The two isolates S44_RUTF and S41_PIFM contained five prophages, named Enterobacteria phage SfI (NC_027339), Enterobacter phage Tyrion (NC_031077), Salmonella phage 118970_sal3 (NC_031940), Escherichia phage P2 (NC_041848), and Enterobacteria phage mEp235 (NC_019708). While the first three of them were intact, Escherichia phage P2 was incomplete, and Enterobacteria phage mEp235 (NC_019708) was questionable. The Salmonella phage 118970_sal3 (NC_031940) was present in all of the isolates in this investigation, with the length of the prophage ranging from 26.4 to 60.3 kbp. The isolate S43_TF only harbored two types of prophages, the Cronobacter phage ENT47670 (NC_019927) and Salmonella phage SSU5 (NC_018843). Both of them were incomplete (Figure 7, Supplementary Table S5). None of the phage sequences carried any AMR gene.

Similarly to the prophage regions, *C. sakazakii* isolates had a clear pattern of plasmid replicons. PlasmidFinder detected a total of 2 distinct plasmid replicons in these genomes. S44_RUTF and S41_PIFM contained two plasmid replicons, IncFII (pCTU2) (FN543095) and pESA2 (CP000784), which were absent in S43_TF (Figure 7, Supplementary Table S6). Moreover, MobileElementFinder detected an insertion sequence from the IS3 family, ISKpn34 (CP008932), in S43_TF (Supplementary Table S7).

### 3.7. Detection of CRSIPR

All the study isolates contained Type I-E CRISPR systems in their genomes. Some CAS-putative and CRISPRs without cas were also detected. CRISPR numbers varied from 20 to 25, while one to three CAS-putatives were found in the genomes (Supplementary Table S8).

## 4. Discussion

It is well known that *Cronobacter* spp. can cause severe infections in neonates and infants through ingestion of contaminated infant formula [39,40,41,42]. In the current study, we isolated two *C. sakazakii* isolates belonging to ST83 (S44_RUTF and S41_PIFM) and one isolate of ST789 (S43_TF), isolated from ready-to-use therapeutic food, powdered infant formula milk, and therapeutic food, respectively. To our knowledge, this is the first report of whole genome analysis of *C. sakazakii* from Bangladesh.

The whole genome analysis revealed that the isolates S44_RUTF, S41_PIFM belong to ST83, one of the most common sequence types associated with disease in infants and children [43]. The ST83 pathovar, along with ST4, ST1, ST8, and ST12, is most frequently found in powdered infant formula marketed in different countries, in powdered infant formula production plants, and in invasive clinical cases such as fatal meningitis and septicemia [1,44,45,46,47]. In particular, *C. sakazakii* ST83 isolates exhibited specific adaptations to persist in powdered infant formula manufacturing facilities and stress-tolerance conditions [48]. This sequence type of *C. sakazakii* has been from environmental and infant formula samples, and was associated with septicemia in infants [40]. In Bangladesh, there is no study on clinical isolates of *C. sakazakii* so far, and therefore, it is not clear which sequence type might be circulating in the country. Detection of *C. sakazakii* ST83 isolates, which are frequently linked to infant and children infection, poses a great threat to infant health. This data suggests further study on clinical cases of infant meningitis and septicemia for the presence of *C. sakazakii*.

In contrast, the *C. sakazakii* isolate S43_TF belongs to a very new sequence type ST789, which has been reported for the first time in 2023 in powdered infant formula and processing environments in China [49]. The current study is the first comprehensive genome analysis of this rare sequence type for the first time. Although until now there is no report of any clinical cases associated with ST789, the presence of an array of virulence genes, with some unique ones, indicates its virulence potential and emphasizes on more comprehensive investigations on this type in infant food products. In particular, the genes *gtrA* and *gtrB*, involved in surface polysaccharide modification, were unique to S43_TF, possibly altering antigenic properties and immune recognition [50]. Five chemotaxis-related genes (*cheB*, *cheR*, *cheW*, *cheY*, and *cheZ*), which may enhance its ability to detect and move toward favorable niches, are a factor linked to increased colonization efficiency [51].

In addition to the unique virulence genes, this ST789 isolate, S43_TF, along with the other two ST83 isolates, revealed a repertoire of virulence-associated genes. All of them possessed multiple genes linked to motility, adhesion, iron acquisition, and stress tolerance. Collectively, these virulence determinants suggest that these *C. sakazakii* isolates have the potential to cause severe infections, particularly in neonates and immunocompromised individuals, in line with previous reports on *C. sakazakii* pathogenesis [1].

Antibiotic resistance in *C. sakazakii* is diverse and depends on the source and geographic location of the strain [52,53,54]. The three *C. sakazakii* isolates from the current study also displayed the presence of a diverse collection of antibiotic resistance genes conferring resistance to cephalosporin, phosphonic acid, glycopeptide, and elfamycin. A few of the genes belonged to antibiotic efflux pumps. While some of the genes were related to resistance to a specific class of antibiotics, the others were linked with resistance to multiple antibiotics. These genes were frequently found in earlier studies [3,8,55,56,57]. Antibiotic overuse in food environments and the presence of several antibiotic resistance operons (*marA*) can favor the development of resistance to different antibiotics in *Cronobacter* spp. [55,58,59]. Another gene, EF-Tu, was detected, which is a member of the elfamycin family and resists antibiotics through target alteration. This gene has evolved to be a multifunctional protein in a wide variety of pathogenic bacteria [60]. However, *glpT*, a commonly found AMR gene in *Cronobacter* spp. that confers resistance to fosfomycin, was not found in any of our study isolates. Additionally, *blaCTX* genes, commonly found in cephalosporin-resistant strains, were also not detected in this study [61,62].

In our study, the ST83 isolates contained two plasmid replicons, IncFIB(pCTU2) and pESA2, both of which are conjugative plasmids [63]. Conjugation in bacteria is very common, and plasmids like pESA2 and pCTU2 in *Cronobacter* spp. generally carry gene clusters responsible for T4SS secretion and pilus assembly [63,64]. In addition, pCTU2 has been observed to carry AMR genes in *Salmonella enterica* [65]. Although both of these plasmids have been reported in *C. sakazakii* strains in previous studies [56,66,67], unlike *Salmonella enterica*, no AMR genes were found to be carried by these plasmids. Two commonly found plasmids related to antibiotic resistance, IncFIB(pCTU1) and IncFIB(pCTU3), were absent in our study. The insertion sequence ISKpn34, detected in the *C. sakazakii* S43_TF strain, is also linked to antibiotic resistance, and commonly reported in *Klebsiella pneumoniae* and other Enterobacteriaceae. There are very few reports of this insertion sequence in *C. sakazakii* isolates [68]. Therefore, the presence of this insertion sequence is noteworthy.

The presence of prophages in bacterial genomes can also confer antibiotic and environmental stress resistance, and additionally, aid in bacterial attachment to their host cells, facilitating their virulence potential as pathogens [69]. Investigation of the genome sequences of the three *C. sakazakii* isolates revealed that each isolate harbored at least one intact phage. Except for Enterobacteria phage SfI (NC_027339) and Escherichia phage P2 (NC_041848), all other prophages detected in this study were reported in *C. sakazakii* isolates obtained from different filth flies [70]. In particular, this is the first report of the detection of Enterobacteria phage SfI (NC_027339) and Escherichia phage P2 (NC_041848) in any *C. sakazakii* isolate. Although the Escherichia phage P2 (NC_041848) is also reported for the first time, it was incomplete in nature. The Salmonella phage 118970_sal3 (NC_031940), the only prophage present in all the study isolates, was intact in nature, and its length was quite small compared to an earlier study [70]. However, this prophage with almost a similar length was also detected in a few studies [8,56]. These studies also showed that Enterobacter phage Tyrion (NC_031077), another intact prophage identified in our ST83 isolates, had almost similar length to the isolates in these studies. Notably, this prophage was identified in *C. sakazakii* isolates collected from PIF manufacturing facilities [8]. Salmonella phage SSU5 (NC_018843), which was found in only S43_TF, was observed in ST1 and ST4 in an earlier study [8]. This phage was also discovered in *Pseudomonas* strains that were found in Michigan State’s coastal waters in the United States. It has been shown to produce siderophores and increase resistance to the heavy metals copper and mercury [71].

Investigating the diversity of CRISPR genes in bacteria is important since these systems can be used to type different microbes. CRISPR and cas genes can be horizontally transferred between strains of the same species as well as across species and genera that are far apart [72]. Additionally, studies on *Pseudomonas aeruginosa* also suggest that Type I and Type II CRISPR-cas systems in bacteria help in escaping mammalian immune systems efficiently [73]. The *C. sakazakii* isolates from the current study contained Type I-E CRISPR systems, frequently found in *C. sakazakii* [74,75,76]. Whether this CRISPR system plays any role in immune evasion is worth investigating. However, another CRISPR system I-F was also found in earlier studies, which was absent in ours [57].

## 5. Conclusions

This study underscores the critical public health relevance of monitoring *C. sakazakii* in PIF. The detection of ST83, a sequence type linked to severe neonatal infections, highlights the persistence of established pathogenic strains in Bangladesh. Genomic insights into virulence factors, antibiotic resistance genes, plasmid diversity, and prophages emphasize the capacity of these bacteria to survive, adapt, and potentially cause severe infections in vulnerable populations. The findings also illustrate the role of MGEs in disseminating resistance, underscoring the need for stringent quality control and surveillance in formula production. Overall, this study provides valuable knowledge for risk assessment, informs targeted preventive strategies, and strengthens efforts to safeguard infant health against *C. sakazakii* contamination.

## Figures and Tables

**Table 1:** Assembly statistics of the *C. sakazakii* isolates obtained from infant formula samples in Bangladesh.

Feature	Details		
Isolate ID	S44_RUTF	S41_PIFM	S43_TF
Sample source	Ready-to-use therapeutic food	Powdered infant formula milk	Therapeutic food
Year of isolation	2024	2024	2024
Genome length (bp)	4,366,115	4,371,145	4,444,405
No. of contigs	106	153	38
GC content (%)	56.98	56.97	56.84
N50 (bp)	86,185	60,688	272,134
L50	17	23	5
Coverage (X)	23.22	19.39	123.1
NCBI accession	NZ_JBNUPD000000000	NZ_JBNPBD000000000	NZ_JBNUPC000000000

**Table 2:** Overview of the features of the annotated genomes.

Feature	Details		
Isolate ID	S44_RUTF	S41_PIFM	S43_TF
CDS	4037	4066	4076
Partial CDS	0	0	0
rRNA	3	5	3
tRNA	62	64	68
tmRNA	1	1	1
Miscellaneous RNA	0	0	0
Repeat Regions	2	2	2
Subsystems	351	351	350
Hypothetical proteins	629	642	700
Proteins with functional assignments	3658	3713	3596
Proteins with EC number assignments	1140	1157	1135
Proteins with GO assignments	929	944	928
Proteins with Pathway assignments	807	820	805

**Table 3:** MLST profiling of the *C. sakazakii* isolates.

Isolate ID	Sequence Type	Locus	Identity	Coverage	Alignment Length	Allele Length	Gaps	Allele
S44_RUTF and S41_PIFM	ST83	atpD	100	100	390	390	0	atpD_19
		fusA	100	100	438	438	0	fusA_16
		glnS	100	100	363	363	0	glnS_19
		gltB	100	100	507	507	0	gltB_41
		gyrB	100	100	402	402	0	gyrB_19
		infB	100	100	441	441	0	infB_15
		pps	100	100	495	495	0	pps_23
S43_TF	ST789	atpD	100	100	390	390	0	atpD_15
		fusA	100	100	438	438	0	fusA_14
		glnS	100	100	363	363	0	glnS_15
		gltB	100	100	507	507	0	gltB_13
		gyrB	100	100	402	402	0	gyrB_22
		infB	100	100	441	441	0	infB_5
		pps	100	100	495	495	0	pps_347

**Table 4:** Number of core, accessory, and unique genes taken from the pan-genome analysis of 21 *C. sakazakii* strains.

Isolate ID	Assembly Accession	No. of Core Gene	No. of Accessory Genes	No. of Unique Genes
*Cronobacter sakazakii* ATCC BAA_894	GCA_000017665.1	3317	749	86
*Cronobacter sakazakii* ES15	GCA_000263215.1	3317	527	44
*Cronobacter sakazakii* SP291	GCA_000339015.1	3317	735	95
*Cronobacter sakazakii* CMCC 45402	GCA_000504545.1	3317	626	270
*Cronobacter sakazakii* CS_931	GCA_003516125.3	3317	652	96
*Cronobacter sakazakii* GZcsf_1	GCA_003955925.1	3317	725	476
*Cronobacter sakazakii* CFSAN068773	GCA_009648895.1	3317	760	95
*Cronobacter sakazakii* 5563_17	GCA_013425995.1	3317	525	49
*Cronobacter sakazakii* C767	GCA_018884085.1	3317	896	0
*Cronobacter sakazakii* C79	GCA_018884105.1	3317	668	138
*Cronobacter sakazakii* C105731	GCA_018884125.1	3317	651	96
*Cronobacter sakazakii* C757	GCA_018884145.1	3317	896	5
*Cronobacter sakazakii* G4023	GCA_020869275.1	3317	855	280
*Cronobacter sakazakii* 70402496	GCA_022648965.1	3317	697	99
*Cronobacter sakazakii* JXES_28	GCA_023805435.1	3317	657	84
*Cronobacter sakazakii* USDA_ARS_USMARC_54664	GCA_025200595.1	3317	571	60
*Cronobacter sakazakii* Crono_589	GCA_025345525.1	3317	642	166
*Cronobacter sakazakii* Crono_684	GCA_025345545.1	3317	735	146
*Cronobacter sakazakii* S41_PIFM	GCF_050311875.1	3317	720	20
*Cronobacter sakazakii* S43_TF	GCF_050409185.1	3317	656	84
*Cronobacter sakazakii* S44_RUTF	GCF_050409205.1	3317	716	9

**Table 5:** Overview of the unique genes found in the study isolates after pan-genome analysis.

Isolate ID	Gene	Function
S44_RUTF	*astE*	Succinylglutamate desuccinylase
	*curA*	NADPH-dependent curcumin reductase
	*nasR*	Nitrate regulatory protein
S41_PIFM	*fdhF_5*	Formate dehydrogenase H
	*pepP*	Xaa-Pro aminopeptidase
S43_TF	*aplIM*	Modification methylase AplI
	*hin*	DNA-invertase hin
	*jefA*	Drug efflux pump JefA
	*xerC_4*	Tyrosine recombinase XerC

**Table 6:** Overview of the antimicrobial resistance genes in the *C. sakazakii* isolates.

Antibiotic Resistance Ontology (ARO) Term	SNP	Drug Class	Resistance Mechanism	S44_RUTF	S41_PIFM	S43_TF
*acrA*		Fluoroquinolone antibiotic, cephalosporin, glycylcycline, penicillin beta-lactam, tetracycline antibiotic, rifamycin antibiotic, phenicol antibiotic, disinfecting agents, and antiseptics	Antibiotic efflux	+	+	+
*adeF*		Fluoroquinolone antibiotic, tetracycline antibiotic	Antibiotic efflux	+	+	+
*cRP*		Macrolide antibiotic, fluoroquinolone antibiotic, penicillin beta-lactam	Antibiotic efflux	+	+	+
*emrB*		Fluoroquinolone antibiotic	Antibiotic efflux	+	+	+
*emrR*		Fluoroquinolone antibiotic	Antibiotic efflux	+	+	+
*H-NS*		Macrolide antibiotic, fluoroquinolone antibiotic, cephalosporin, penicillin beta-lactam, tetracycline antibiotic	Antibiotic efflux	+	+	+
*kpnE*		Macrolide antibiotic, aminoglycoside antibiotic, cephalosporin, tetracycline antibiotic, peptide antibiotic, rifamycin antibiotic, disinfecting agents, and antiseptics	Antibiotic efflux	+	+	+
*kpnF*		Macrolide antibiotic, aminoglycoside antibiotic, cephalosporin, tetracycline antibiotic, peptide antibiotic, rifamycin antibiotic, disinfecting agents, and antiseptics	Antibiotic efflux	+	+	+
*msbA*		Nitroimidazole antibiotic	Antibiotic efflux	+	+	+
*qacG*		Disinfecting agents and antiseptics	Antibiotic efflux	−	−	+
*qacJ*		Disinfecting agents and antiseptics	Antibiotic efflux	+	+	−
*rsmA*		Fluoroquinolone antibiotic, diaminopyrimidine antibiotic, phenicol antibiotic	Antibiotic efflux	+	+	+
*marA*		Fluoroquinolone antibiotic, monobactam, carbapenem, cephalosporin, glycylcycline, penicillin beta-lactam, tetracycline antibiotic, rifamycin antibiotic, phenicol antibiotic, disinfecting agents, and antiseptics	Antibiotic efflux, reduced permeability to antibiotics	+	+	+
*csa1*		Cephalosporin	Antibiotic inactivation	+	+	−
*fosA8*		Phosphonic acid antibiotic	Antibiotic inactivation	+	+	+
*vanG*		Glycopeptide antibiotic	Antibiotic target alteration	+	+	+
*eF-Tu*	R234F	Elfamycin antibiotic	Antibiotic target alteration	+	+	+
*pbp3*	D350N, S357N	Penicillin-binding protein mutations conferring resistance to beta-lactam antibiotics	Antibiotic target alteration	+	+	+
*acrAB-tolC with marR mutations*	S3N	Fluoroquinolone antibiotic, cephalosporin, glycylcycline, penicillin beta-lactam, tetracycline antibiotic, rifamycin antibiotic, phenicol antibiotic, disinfecting agents, and antiseptics	Antibiotic target alteration, antibiotic efflux	+	+	+

Here, + = Present, − = Absent.

## Data Availability

Data available in a publicly accessible repository, National Center for Biotechnology Information (NCBI). Accession numbers are NZ_JBNUPD000000000 (S44_RUTF), NZ_JBNPBD000000000 (S41_PIFM), and NZ_JBNUPC000000000 (S43_TF).

## Supplementary Materials

The following supporting information can be downloaded at: https://www.mdpi.com/article/10.3390/pathogens14121220/s1, Table S1: Metadata of the genomes (n = 18) downloaded from BV-BRC for pan-genome analysis; Table S2: Average nucleotide identity (ANI) among the study isolates (n = 3) and the isolates downloaded from BV-BRC (n = 18) for pan-genome analysis; Table S3: Antimicrobial resistance (AMR) genes identified in the study genomes using CARD; Table S4: Overview of the outcome of the study isolates using PathogenFinder; Table S5: Prophage regions identified in the study genomes using PHASTEST; Table S6: Plasmid replicons identified in the study genomes using PlasmidFinder; Table S7: Mobile genetic element (MGE) detected in S43_TF; Table S8: CRISPR systems identified in the study genomes using CRISPRCasFinder.
